# Prevalence of primary outcome changes in clinical trials registered on ClinicalTrials.gov: a cross-sectional study

**DOI:** 10.12688/f1000research.3784.1

**Published:** 2014-03-26

**Authors:** Sreeram Ramagopalan, Andrew P. Skingsley, Lahiru Handunnetthi, Michelle Klingel, Daniel Magnus, Julia Pakpoor, Ben Goldacre

**Affiliations:** 1 Medical Research Council Functional Genomics Unit and Department of Physiology, Anatomy and Genetics, University of Oxford, Oxford, OX1 3PT, UK; 2 London School of Hygiene and Tropical Medicine, London, WC1E 7HT, UK

## Abstract

**Background**: An important principle in the good conduct of clinical trials is that a summary of the trial protocol, with a pre-defined primary outcome, should be freely available before the study commences. The clinical trials registry ClinicalTrials.gov provides one method of doing this, and once the trial is registered, any changes made to the primary outcome are documented. The objectives of this study were: to assess the proportion of registered trials on ClinicalTrials.gov that had the primary outcome changed; to assess when the primary outcome was changed in relation to the listed study start and end dates and to assess whether the primary outcome change had any relation to the study sponsor.

**Methods**: A cross-sectional analysis of all interventional clinical trials registered on ClinicalTrials.gov as of 25 October 2012 was performed. The main outcome was any change made to the initially listed primary outcome and the time of the change in relation to the trial start and end date.

**Findings**: Our analysis showed that 28229 of 89204 (31.7%) registered studies had their primary outcome changed.  Industry funding was associated with all primary outcome changes, odds ratio (OR)= 1.36, 95% confidence interval (CI)=1.31-1.41, p<0.001; with primary outcome changes after study start date OR=1.37, 95% CI=1.32-1.42, p<0.001; with primary outcome changes after primary completion date OR=1.84, 95% CI=1.75-1.94, p<0.001 and with primary outcome changes after study completion date OR=1.82, 95% CI=1.73-1.91, p<0.001.

**Conclusions** A significant proportion of interventional trials registered on ClinicalTrials.gov have their primary outcomes altered after the listed study start and completion dates. These changes are associated with funding source.

## Introduction

Clinical trials provide the principal method with which to assess the effectiveness of therapeutic strategies ^1^. An important principle in the good conduct of clinical trials is that a summary of the trial protocol, with a pre-defined primary outcome, should be freely available before the study commences ^1^. In February 2000, the United States (US) Food and Drug Administration (FDA) created an online clinical trials registry named ClinicalTrials.gov ^2^. From 2005, the International Committee of Medical Journal Editors (ICMJE) required that clinical trials should be listed in a clinical trial registry to qualify for publication ^3^. The registration of a clinical trial usually involves reporting informationon 20 items proposed by the World Health Organization (WHO) registration advisory group, including the primary outcome of the study ^4^. One reason for the creation of this registry was to help to reduce the risk of selective reporting of outcomes that had been previously identified. For example, a cohort study using protocols and published reports of randomized trials approved by the Scientific-Ethical Committees for Copenhagen and Frederiksberg in Denmark in 1994–1995 found that 62% of trials had at least one primary outcome that was changed, introduced, or omitted ^5^. A more recent study looking at trials that were registered on trial websites (such as ClinicalTrials.gov), found that 31% of trials displayed some evidence of discrepancies between the outcomes registered and the outcomes published ^6^. Therefore, even trial registration may not be a complete barrier to selective outcome reporting.

ClinicalTrials.gov tracks all changes made to registered protocols. The objectives of this study were: to assess the proportion of registered trials on ClinicalTrials.gov that had the primary outcome changed; to assess when the primary outcome was changed in relation to the listed study start and end dates and to assess whether the primary outcome change had any relation to the study sponsor.

## Methods

### Data source

ClinicalTrials.gov is a publicly available trial registry and results database developed and maintained by the US National Library of Medicine on behalf of the US National Institutes of Health.

### Study sample

We wrote scripts in R to download all interventional clinical studies registered with ClinicalTrials.gov as of 25 October 2012. Data from the ‘tabular view’ for all studies ( *e.g*. http://clinicaltrials.gov/ct2/show/record/NCT00548405?term=alemtuzumab+multiple+sclerosis&rank=2) were downloaded, and the information was automatically extracted from each field and used to populate a spreadsheet for analysis. This spreadsheet is available in *figshare* ( doi: 10.6084/m9.figshare.967827). We downloaded data from all interventional studies registered with ClinicalTrials.gov as of 25 October 2012 preventing any bias in study selection (please contact the corresponding author for details on the scripts).

The following information was collected from each study: study registration date (the date the study is registered with ClinicalTrials.gov); the study start date (defined as the date that enrollment to the protocol begins); the primary completion date (defined as the anticipated or actual date the final subject was examined or received an intervention for the purposes of final collection of data for the primary outcome, whether the clinical trial concluded according to the pre-specified protocol or was terminated); study completion date (defined as the final date on which data was collected); original primary outcome (defined as a specific key measurement(s) or observation(s) used to measure the effect of experimental variables in a study) and date submitted; current primary outcome and date submitted; study phase (phase of investigation as defined by the US FDA), data monitoring committee (whether an independent group of scientists has been appointed to monitor the safety and the scientific integrity of a human research intervention, and to make recommendations to the sponsor regarding the termination of the trial for efficacy, for harm or for futility); study sponsor (defined as the primary organization that oversees the implementation of the study and is responsible for data analysis) and collaborators (defined as other organizations (if any) providing support, including funding, design, implementation, data analysis and reporting).

For studies to be included in this analysis a primary outcome had to be registered. A study was classified as not having a primary outcome changed if the original primary outcome was listed as ‘same as current’. For a subset of interventional trials, specifically those that had ‘multiple sclerosis’ or ‘diabetes’ in the title, we looked for primary outcomes that had a significant change, as the authors have experience in these fields. We defined a discrepancy between primary outcomes if the two were clearly different ( *e.g.* in one study the primary outcome was changed from ‘expanded disability status scores assessed every 12 weeks’ to ‘annualized relapse rate’). Two authors (SVR and JP) independently assessed whether or not the studies had significant changes; then they met to compare results. For any discrepancies they met with another author (LH) and a consensus was reached. The kappa statistic for agreement between the first two observers was 0.87.

ClinicalTrials.gov stores funding organization information using two data elements: lead sponsor (defined as the organization or person who oversees the clinical study and is responsible for analyzing the study data) and collaborator (defined as an organization other than the sponsor that provides support for a clinical study). We derived probable funding source from the lead sponsor and collaborator fields using the following algorithm: if the lead sponsor and any collaborators were from industry and no non-industry sponsors or collaborators were listed, then the study was categorized as industry funded; if the lead sponsor and/or collaborator included industry and non-industry sources, then the study was categorized as mixed; finally if the lead sponsor and any collaborators were not from industry and no industry sponsors or collaborators were listed, then the study was categorized as non-industry funded. Two authors (SVR and JP) independently appraised the funding status of all studies; then they met to compare results. For any discrepancies they consulted another author (LH) and a final consensus was reached. The kappa statistic for agreement between the first two observers was 0.76.

***Statistical analysis.*** We used logistic regression to calculate odds ratios (ORs) and 95% confidence intervals (95% CI) for comparisons between outcome changed and non-outcome changed groups, using registration date and funding source as explanatory variables. In a full model, we included study phase and data monitoring committee as additional explanatory variables. The *p*-values <0.05 were interpreted as significant. Statistical analyses were conducted using the STATA 12.0.0 software.

Data set of primary outcome changes in interventional clinical trials registered on ClinicalTrials.org All clinical studies classified as ‘interventional studies’ registered with ClinicalTrials as of 25 October 2012 are shown. The following information was collected from each study: study registration date; the study start date; the primary completion date; study completion date; original primary outcome and date submitted; current primary outcome and date submitted; study phase, data monitoring committee; study sponsor and collaborators.

## Results

### Study sample

As of 25 October 2012, 97,668 interventional trials were registered with ClinicalTrials.gov. These trials were registered between 1999 and 2012; the distribution of registrations by year is shown in Table 1. There were 46,803 (52.5%) trials classed as non-industry funded, 11,986 (13.4%) as mixed and 30,415 (34.1%) as industry funded ( Table 1). There were 39,992 studies registered as completed.

**Table 1.** Number of interventional studies registered on ClinicalTrials.gov by year, funding status and primary outcome change.

	Publicly funded		Mixed funding		Industry funded	
Year	Unchanged	Changed	Unchanged	Changed	Unchanged	Changed
**1999**	286	142	19	1	3	6
**2000**	88	91	17	6	1	0
**2001**	85	141	6	1	3	7
**2002**	127	186	26	22	137	94
**2003**	139	333	15	20	125	108
**2004**	152	460	17	30	250	248
**2005**	2017	2775	767	860	1817	1717
**2006**	1519	2402	437	696	1223	1897
**2007**	2055	2798	563	885	1168	2473
**2008**	4761	1304	1170	360	2882	1750
**2009**	5042	1146	1337	314	2742	1254
**2010**	5609	890	1412	254	2868	850
**2011**	6184	548	1458	146	3367	463
**2012**	5241	282	1088	59	2752	210

### Primary outcome changes

We found that 28,129 studies (31.7% of 89,204) had their primary outcome changed. Funding source and registration year were associated with trials that had their primary outcome changed ( Table 2). Restricting analyses to completed trials (39,992 trials) did not materially change these associations (mixed funding odds ratio (OR)=1.02; 95% confidence interval (CI)=0.95–1.10, p=0.63; industry funding OR=1.30, 95% CI=1.24–1.36, p<0.001).

**Table 2.** Association of funding status and registration year with primary outcome change for a) all primary outcome changes, b) primary outcome changes after listed study start date, c) primary outcome changes after listed primary completion date on ClinicalTrials.gov and d) primary outcome changes after listed study completion date on ClinicalTrials.gov. OR=odds ratio.

	a) OR (95% confidence interval)	*p* value	b) OR (95% confidence interval)	*p* value	c) OR (95% confidence interval)	*p* value	d) OR (95% confidence interval)	*p* value
**Funding**								
Non-industry	1		1		1		1	
Mixed	1.01 (0.96–1.06)	0.33	1.01 (0.96–1.07)	0.60	1.08 (1.00–1.17)	0.04	1.10 (1.02–1.18)	0.019
Industry	1.36 (1.31–1.41)	<0.001	1.37 (1.32–1.42)	<0.001	1.84 (1.75–1.94)	<0.001	1.82 (1.73–1.91)	<0.001
**Registration** **Year**								
1999	1		1		1		1	
2000	1.90 (1.36–2.66)	<0.001	1.62 (1.15–2.28)	0.006	1.14 (0.71–1.82)	0.59	1.41 (0.79–2.53)	0.25
2001	3.26 (2.36–4.50)	<0.001	3.02 (2.16–4.21)	<0.001	1.79 (1.13–2.82)	0.012	1.95 (1.11–3.45)	0.021
2002	1.92 (1.49–2.48)	<0.001	1.62 (1.25–2.10)	<0.001	1.69 (1.16–2.47)	0.007	1.81 (1.18–2.77)	0.007
2003	3.13 (2.45–4.00)	<0.001	2.74 (2.13–3.53)	<0.001	2.37 (1.64–3.42)	<0.001	2.74 (1.80–4.17)	<0.001
2004	3.22 (2.56–4.06)	<0.001	2.74 (2.17–3.47)	<0.001	2.37 (1.67–3.36)	<0.001	2.59 (1.73–3.87)	<0.001
2005	2.17 (1.78–2.65)	<0.001	1.86 (1.51–2.28)	<0.001	2.61 (1.90–3.58)	<0.001	2.02 (1.39–2.94)	<0.001
2006	2.91 (2.38–3.56)	<0.001	2.47 (2.01–3.04)	<0.001	2.29 (1.67–3.15)	<0.001	2.24 (1.54–3.26)	<0.001
2007	3.03 (2.48–3.70)	<0.001	2.41 (1.96–2.96)	<0.001	1.61 (1.17–2.21)	0.003	2.00 (1.38–2.90)	<0.001
2008	0.71 (0.58–0.87)	0.001	0.58 (0.47–0.71)	<0.001	0.68 (0.50–0.94)	0.019	1.10 (0.76–1.60)	0.62
2009	0.55 (0.45–0.68)	<0.001	0.43 (0.35–0.53)	<0.001	0.52 (0.38–0.71)	<0.001	0.89 (0.61–1.29)	0.54
2010	0.38 (0.31–0.46)	<0.001	0.27 (0.22–0.34)	<0.001	0.32 (0.23–0.45)	<0.001	0.56 (0.39–0.83)	0.003
2011	0.20 (0.16–0.24)	<0.001	0.12 (0.10–0.15)	<0.001	0.14 (0.10–0.20)	<0.001	0.24 (0.16–0.36)	<0.001
2012	0.11 (0.09–0.14)	<0.001	0.06 (0.04–0.07)	<0.001	0.16 (0.11–0.23)	<0.001	0.27 (0.18–0.42)	<0.001

### Primary outcome changes in relation to study start date

The dates of primary outcome changes were first compared in relation to the registered study start date. This analysis showed that 26,457 studies (30.3% of 87384 studies with a registered start date) had their primary outcome changed after the study had supposedly started. Funding source and registration year were associated with trials that had their primary outcome changed after the study start date ( Table 2). Restricting analyses to completed trials (39236 trials) did not affect the associations with funding (mixed funding OR=1.01; 95% CI=0.94–1.09, p=0.70; industry funding OR=1.29, 95% CI=1.23–1.35, p<0.001).

### Primary outcome changes in relation to study end date

The dates of primary outcome changes were then compared to the listed study end dates. The results showed that 11,834 studies (30.4% of 38,974 studies with a registered primary completion date) had their primary outcome changed after the primary completion date and 10,623 (26.2% of 40,615 studies with a registered study completion date) studies had their primary outcome changed after the study completion date. Funding source and registration year were associated with trials that had their primary outcome changed after either study completion dates ( Table 2). Restricting analyses to completed trials (32,124 trials with a primary completion date and 35,719 trials with a study completion date) rendered the associations with mixed funding non-significant (primary completed trials mixed funding OR=1.10; 95% CI=0.99–1.19, p=0.057; completed trials mixed funding OR=1.08, 95% CI=0.99–1.17, p=0.085); the associations with industry funding remained (primary completed trials industry funding OR=1.94; 95% CI=1.83–2.05, p<0.001; completed trials industry funding OR=1.81, 95% CI=1.72–1.91, p<0.001).

### Inclusion of study phase and use of a data monitoring committee

In a full model we included study phase and the use of a data monitoring committee (DMC) as additional explanatory variables. There were 48,471 trials that provided information for all four variables, 47,748 trials that also had a study start date, 23,080 that also had a primary completion date and 21,747 that also had a study completion date. Funding source was associated to all primary outcome changes, primary outcome changes after listed study start date and primary outcome changes after listed primary completion date following adjustment for study phase and presence of a DMC ( Table 3). Study phase and use of a DMC were also associated to primary outcome changes. All associations remained similar when restricting to completed trials, although the associations of mixed funding with all primary outcome changes and primary outcome changes after study start date became non-significant.

**Table 3.** Association of funding status, registration year, use of a data monitoring committee and study phase with a) all primary outcome changes, b) primary outcome changes after listed study start date *, c) primary outcome changes after listed primary completion date on ClinicalTrials.gov and d) primary outcome changes after listed study completion date on ClinicalTrials.gov*. OR=odds ratio.

	a) OR (95% confidence interval)	*p* value	b) OR (95% confidence interval)	*p* value	c) OR (95% confidence interval)	*p* value	d) OR (95% confidence interval)	*p* value
**Funding**								
Non-industry	1		1		1		1	
Mixed	1.07 (1.00–1.15)	0.04	1.08 (1.01–1.16)	0.024	1.01 (0.91–1.12)	0.84	1.08 (0.97–1.19)	0.15
Industry	1.65 (1.56–1.73)	<0.001	1.69 (1.60–1.78)	<0.001	1.73 (1.61–1.86)	<0.001	1.74 (1.62–1.88)	<0.001
**Registration** **Year**								
1999	1		1		1		1	
2000	1.38 (0.82–2.34)	0.23	1.35 (0.80–2.29)	0.26	1.15 (0.63–2.08)	0.65	0.95 (0.47–1.92)	0.88
2001	2.73 (1.57–4.72)	<0.001	2.67 (1.54–4.64)	<0.001	3.00 (1.65–5.44)	<0.001	2.19 (1.11–4.31)	0.024
2002	2.69 (1.73–4.19)	<0.001	2.61 (1.68–4.07)	<0.001	2.55 (1.57–4.13)	<0.001	1.82 (1.06–3.14)	0.03
2003	7.01 (4.55–10.8)	<0.001	6.76 (4.39–10.43)	<0.001	5.92 (3.68–9.50)	<0.001	4.27 (2.57–7.12)	<0.001
2004	8.80 (5.76–13.45)	<0.001	8.56 (5.60–13.09)	<0.001	5.99 (3.81–9.41)	<0.001	4.88 (2.97–8.02)	<0.001
2005	12.33 (8.61–17.66)	<0.001	11.64 (8.13–16.69)	<0.001	5.82 (3.94–8.59)	<0.001	4.69 (3.04–7.24)	<0.001
2006	16.34 (11.37–23.47)	<0.001	14.91 (10.38–21.43)	<0.001	4.22 (2.86–6.23)	<0.001	3.84 (2.49–5.93)	<0.001
2007	5.26 (3.70–7.47)	<0.001	4.73 (3.32–6.73)	<0.001	2.21 (1.50–3.25)	<0.001	1.82 (1.18–2.81)	0.006
2008	1.11 (0.78–1.58)	0.56	1.01 (0.71–1.43)	0.97	0.93 (0.63–1.36)	0.70	0.94 (0.61–1.45)	0.79
2009	0.85 (0.60–1.21)	0.36	0.74 (0.52–1.05)	0.096	0.70 (0.48–1.04)	0.076	0.78 (0.50–1.20)	0.26
2010	0.58 (0.41–0.83)	0.002	0.47 (0.33–0.67)	<0.001	0.43 (0.29–0.64)	<0.001	0.49 (0.31–0.76)	0.001
2011	0.30 (0.21–0.43)	<0.001	0.21 (0.15–0.30)	<0.001	0.16 (0.10–0.24)	<0.001	0.17 (0.10–0.27)	<0.001
2012	0.16 (0.11–0.23)	<0.001	0.09 (0.06–0.13)	<0.001	0.22 (0.14–0.36)	<0.001	0.24 (0.15–0.41)	<0.001
**Phase**								
0	1		1		1		1	
1	1.37 (1.05–1.78)	0.02	1.37 (1.04–1.80)	0.027	1.05 (0.68–1.63)	0.83	1.10 (0.68–1.76)	0.71
2	1.53 (1.18–1.99)	0.001	1.54 (1.17–2.02)	0.002	1.67 (1.08–2.58)	0.02	1.70 (1.06–2.73)	0.028
3	1.63 (1.26–2.13)	<0.001	1.62 (1.23–2.13)	0.001	2.22 (1.43–3.44)	<0.001	2.22 (1.38–3.57)	0.001
4	1.23 (0.94–1.60)	0.125	1.25 (0.95–1.65)	0.109	2.06 (1.33–3.20)	0.001	2.00 (1.25–3.22)	0.004
**DMC**								
No	1		1		1		1	
Yes	1.17 (1.12–1.23)	<0.001	1.13 (1.07–1.19)	<0.001	0.82 (0.77–0.88)	<0.001	0.81 (0.75–0.86)	<0.001

### Significant primary outcome changes in multiple sclerosis interventional trials

A subset of interventional trials in multiple sclerosis was investigated to assess the actual change in primary outcome. Fifty out of 422 (11.9%) registered trials were deemed to have a significant change in the primary outcome, 49 out of 416 (11.8%) were changed after the study start date, 32 out of 187 (17.1%) were changed after the primary completion date and 26 out of 191 (13.6%) were changed after the study completion date. After adjusting for study registration year, industry funding was associated with all primary outcome changes (OR=4.92, 95% CI 2.12–11.41, p<0.001), primary outcome changes after listed study start date (OR=4.75, 95% CI 2.04–11.05, p<0.001), primary outcome changes after listed primary completion date (OR=14.3, 95% CI 1.87–109.3, p=0.01), and primary outcome changes after listed study completion date (OR=14.52, 95% CI 1.90–111.06, p=0.01). These associations remained also when adjusting for presence of DMC and study phase and when looking at completed trials.

### Significant primary outcome changes in diabetes interventional trials

A subset of interventional trials in diabetes was also investigated to assess the actual change in primary outcome. Two hundred and forty eight out of 2836 (8.7%) registered trials were deemed to have a significant change in the primary outcome, 225 out of 2786 (8.1%) were changed after the study start date, 158 out of 1475 (10.7%) were changed after the primary completion date and 147 out of 1561 (9.4%) were changed after the study completion date. After adjusting for study registration year, industry funding was associated to all primary outcome changes (OR=1.76, 95% CI 1.31–2.36, p<0.001), primary outcome changes after listed study start date (OR=1.75, 95% CI 1.28–2.38, p<0.001), primary outcome changes after listed primary completion date (OR=1.83, 95% CI 1.20–2.77, p=0.005), and primary outcome changes after listed study completion date (OR=2.63, 95% CI 1.68–4.11, p<0.001). These associations remained also when adjusting for presence of DMC and study phase and when looking at completed trials.

## Discussion

### Summary

We assessed the proportion of primary outcome changes for interventional trials registered on ClinicalTrials.gov from 1999 to 2012, when these changes occurred and whether these changes related to funding source. Industry funding was associated with primary outcome changes. These associations remained even after adjusting for the phase of the study and the presence of a DMC. The changes appeared to peak in the period between 2004 and 2007. This may suggest that a lower number of trials were registered before this time period and perhaps that there was not enough time to accrue for changes to be made in more recently registered trials (or that the changes made to the protocol decrease over time). When looking at significant primary outcome changes, the proportion of trials with their outcome changed was much less than all changes; nevertheless, industry funding was still associated with significant primary outcome changes.

There are many reasons for departures from the initial study protocol. Authors should identify and explain any such changes, however no such information is given on ClinicalTrials.gov and thus we have no explanation for our results. The results obtained here may suggest that industry funded trials are more diligent in reporting changes to protocols. Indeed, the primary outcome measure data element was not available in ClinicalTrials.gov until late 2004 and it was considered an optional data element until December 2012. There are implications of the data reported here for medical journals, reviewers and drug approval agencies. ****

### Limitations of this study

A limitation for our study is the scope of the data. We used data from only one trial website, and thus the generalisability to other registration sites is unclear. We have also assumed the data entered regarding study start and completion dates were accurate. We have no information on the reasons for the changes being made. We also did not assess the significance of the primary outcome change for the vast majority of changes; the sub-studies in multiple sclerosis and diabetes suggested that significant changes may only occur in approximately 8% of all registered trials, but this may or may not be applicable to trials for other disorders. Some primary outcome changes may be typographical/semantic and may not reflect actual changes to the nature of the outcome (although one would expect these to occur equally regardless of funding source).

### Background from other studies

Previous reports have not investigated changes to a primary outcome as entered on a registration website, but they have compared trial protocols to published studies. Four previous studies have shown that in 47–74% of studies the primary outcome stated in a protocol was the same as in a subsequent publication; between 13 and 31% of primary outcomes specified in the protocol were omitted in the publication and between 10 and 18% of reports introduced a primary outcome in the publication that was not specified in the protocol ^7^. The proportion of primary outcome changes that we found was perhaps lower than that found previously, but this may be due to the different methodology used in this study. The relationship between protocol changes and funding has not been thoroughly investigated. Chan and colleagues found that 61% of the 51 trials with major discrepancies between the study protocol and publication were funded solely by industry sources compared to 49% of the 51 trials without discrepancies ^5^.

## Conclusions

Primary outcome changes are made to study protocols registered on ClinicalTrials.gov and these changes are associated with funding source.

## Data availability

*figshare:* Data set of primary outcome changes in interventional clinical trials registered on ClinicalTrials.org ^8^. http://dx.doi.org/10.6084/m9.figshare.967827
