# Supplementary material for: Prevalence of primary outcome changes in clinical trials registered on ClinicalTrials.gov: a cross-sectional study
Source: F1000Res. 2014 Mar 26;3:77. [Version 1] doi: 10.12688/f1000research.3784.1 (PMC4032105; doi:10.12688/f1000research.3784.1)
Supplement: Peer review history

# Peer review: Referee response for version 1

**DOI:** 10.5256/f1000research.4053.r4262

**Published:** 2014-05-09

**Authors:** Steven A Julious

**Affiliations:** 1 Medical Statistics Group, University of Sheffield, Sheffield, UK

This is an interesting report with some interesting results. Upon reading the report it did lead to the thinking of possible confounding factors which could explain some of the differences if not all. 

Some of the confounding factors could not be easily investigated in the current data set and I will consider these first.

The first is whether the treatment being investigated in the trial is a licensed or unlicensed therapy. Though in theory "do-able" the authors can be forgiven for not investigating this. However, could it be proposed that a trial of a new unlicensed treatment where knowledge is emerging - and being published as the trial is ongoing - is more likely to have an endpoint changed than a licensed treatment with known properties?

The other consideration is therapeutic area. The authors to a degree looked into this by investigating two sub populations of MS and diabetes trials but it could be that certain therapeutic areas predominate in certain sectors?

One final consideration is if the endpoint change was pre-planned in some way in some form of adaptive design. 

With respect to confounders which could be investigated the main consideration is study duration. Might a study that has gone on longer be more likely to have an endpoint change? This could be legitimate if, as mentioned previously this change is due to published work becoming available. It could also be (maybe) legitimate if, for completed studies, it has dragged on for an age with no chance to reach the target sample size. So to a degree there is a case of fitting tailoring the study to what can be answered with the sample size. The interaction between study duration and funder would also be interesting (as well as just allowing in the model)

There are two markers in the analysis which do suggest study duration may impact on endpoint change. The first is study registration year and the other is phase of development. In the case of phase of development the effect seems to be the reverse, it could be argued, to what one would expect. Early phase trials are learning trials not confirming trials. A consequence is the endpoints may change to reflect the properties of a treatment an investigator is learning about in an ongoing manner.  It would be interesting with study duration in the model how it would effect the comparison of phase of development

Following on from the point in the previous paragraph therefore an analysis of just trials which are Phase III would be of interest. These trials should have everything set and pre-specified in terms of analysis

One final point about the work is that I could not see mention of a protocol for the audit itself undertaken for the paper. In particular what the pre-specified primary endpoint and analysis population was. It is not the convention for audits to do this but it would have been optimal given the subject matter.

In summary therefore it would be optimal to comment on the potential limitations of the work and to undertake the suggested analyses. However, the work itself is of interest and will almost certainly feed debate.

I have read this submission. I believe that I have an appropriate level of expertise to confirm that it is of an acceptable scientific standard.

# Peer review: Referee response for version 1

**DOI:** 10.5256/f1000research.4053.r4266

**Published:** 2014-04-23

**Authors:** Deborah Korenstein

**Affiliations:** 1 General Internal Medicine, Mount Sinai Hospital, New York, NY, USA

This cross-sectional study of trials registered on Clinicaltrials.gov found that changes in primary outcome are common and are associated with industry funding. The study was appropriately done and reported. The reasons for the changes in primary outcomes and the explanation for the association with funding source are not clear, but it seems likely that changes to the primary outcome might be important for the sound interpretation of results.

I have read this submission. I believe that I have an appropriate level of expertise to confirm that it is of an acceptable scientific standard.

# Peer review: Referee response for version 1

**DOI:** 10.5256/f1000research.4053.r4267

**Published:** 2014-04-07

**Authors:** Janet Wale

**Affiliations:** 1 Cochrane Collaboration Consumer Network, Brunswick, VIC, Australia

This is a well written, competently undertaken, cross-sectional analysis. The study finds that changes to primary outcomes listed in a trials register, at any stage of the trial, are clearly associated with industry funding. Studying the extent of the changes in two disease areas is a practical way used by the authors to assess the changes and their significance (extensive in some 8%).

The authors point out that there are many reasons for changes from the initial study protocol. No information is given on ClinicalTrials.gov on the reasons for changes and thus they have no explanation for their results. The authors suggest that industry funded trials are more diligent in reporting changes to protocols.

This is a possible area for change which has been provided in this article.

I have read this submission. I believe that I have an appropriate level of expertise to confirm that it is of an acceptable scientific standard.
